# A multi-stakeholder multicriteria decision analysis for implantable medical devices assessment in China

**DOI:** 10.3389/frhs.2025.1650709

**Published:** 2025-08-29

**Authors:** Yizhou Xu, Junjie Wan, Bin Wan, Haixia Ding

**Affiliations:** ^1^ School of Pharmacy, Nanjing Medical University, Nanjing, Jiangsu, China; ^2^ School of International Pharmaceutical Business, China Pharmaceutical University, Nanjing, Jiangsu, China; ^3^ Department of Health Insurance Management, The First Affiliated Hospital with Nanjing Medical University, Nanjing, Jiangsu, China

**Keywords:** medical devices, multicriteria decision analysis, discrete choice experiment, technology assessment, preference, China

## Abstract

**Objectives:**

This study aims to develop a standardized multicriteria decision analysis (MCDA) framework tailored for implantable medical devices in China, addressing the challenges of inconsistent evaluation processes under China's evolving healthcare financing policies.

**Methods:**

A mixed-methods design combining a discrete choice experiment (DCE) and MCDA was employed. Six criteria (clinical effectiveness, clinical safety, innovation, disease severity, implementation capacity, and cost) were identified through literature reviews and expert consultations. A DCE survey with 540 multi-stakeholder participants (decision-makers, HTA experts, clinicians, hospital administrators, and citizens) was conducted to derive criterion weights using mixed logit models. The framework was validated through a real-world case study assessing endoscopic linear staplers.

**Results:**

Clinical safety (35.45%) and cost (27.94%) emerged as the most critical criteria, followed by implementation capacity (16.56%) and clinical effectiveness (15.07%). Innovation (2.54%) and disease severity (2.44%) received minimal weight. The MCDA application demonstrated high inter-rater consistency (CV < 0.25).

**Conclusions:**

This study proposes a transparent, stakeholder-driven framework for evaluating implantable medical devices, specifically designed to support China's healthcare policies. The framework ensures that healthcare decisions are grounded in clinical effectiveness, safety, and long-term economic viability.

## 1 Introduction

China's basic medical insurance program has achieved broad population coverage [over 95% as of 2023 (1)], significantly improving healthcare accessibility and financial risk protection for citizens. The nationwide rollout of DRG/DIP prospective payment systems (2) has mandated stricter cost containment and value-based resource allocation across healthcare providers. By replacing fee-for-service with bundled payments and retrospective utilization reviews, these reforms align provider incentives with predefined budget frameworks while maintaining clinical quality standards.

Medical devices (MDs), defined by the World Health Organization as “health technologies for disease diagnosis, treatment or rehabilitation,” (3) play a pivotal role in China's healthcare delivery system. This study focuses on implantable high-value MDs (e.g., cardiac pacemakers, orthopedic prostheses, vascular stents) that constitute major expenditure items in national health insurance funds. Within China's universal healthcare coverage framework, the fragmented medical consumables market presents dual challenges: (a) insurance fund sustainability risks from unregulated price-quality variations, and (b) inequitable patient access due to regional procurement disparities.

The National Healthcare Security Administration (NHSA) has implemented strategic purchasing mechanisms through policies like the Volume-Based Procurement Implementation Plan and dynamic adjustment rules for medical insurance payment standards (4). These initiatives mandate value-based assessment of MDs incorporating clinical efficacy, cost-effectiveness, and budget impact analysis (BIA). Nevertheless, the absence of unified Health Technology Assessment (HTA) guidelines has resulted in two systemic issues: (a) Provider-level evaluation criteria disproportionately weighted (60%–70%) (5) on procurement costs rather than long-term clinical outcomes; (b) Insufficient integration of real-world evidence (RWE) from national insurance claim databases (6).

The inclusion of MDs in healthcare reimbursement schemes necessitates comprehensive evaluation given their substantial financial implications and clinical significance, requiring systematic balancing among three core dimensions: clinical effectiveness, safety profile, and cost-effectiveness (7). Previous studies have underscored both the challenges and critical importance of integrating multi-stakeholder perspectives in medical technology assessments (8).

To overcome these methodological challenges, multi-criteria decision analysis (MCDA) has gained prominence as an evidence-based decision support tool (9, 10). The MCDA framework enables structured decision-making through decomposition of complex evaluations into well-defined criteria, followed by systematic weighting and scoring based on their predetermined importance hierarchy. This methodology has been increasingly adopted in health technology assessment (HTA) systems to enhance decision transparency, maintain evaluative consistency, and improve systemic adaptability within evolving healthcare contexts.

Global implementations of MCDA for medical devices vary significantly based on healthcare system structures, policy priorities, and stakeholder landscapes. Queensland Health's MCDA model reflects Australia's universal healthcare goals through its focus on clinical benefit, cost-effectiveness, and equity of access, with the notable inclusion of explicit criteria for “implement capacity” (e.g., workforce training needs)—a dimension less emphasized in market-driven systems (7). Egypt's recent MCDA tool for implantable devices, by contrast, prioritizes technical characteristics and country of origin, which diverges from Western focus on clinical outcomes (9). These international examples highlight that MCDA frameworks are inherently context-specific, requiring adaptation to local policy objectives and healthcare financing models.

This study aims to develop a multi-criteria decision analysis framework to address the following questions: (a) What are the core criteria for evaluating implantable medical devices in China, considering the priorities of diverse stakeholders? (b) How do stakeholder preferences for these criteria differ, and what weights should be assigned to reflect China's policy goals? (c) Can a stakeholder-driven MCDA framework improve the consistency and transparency of device evaluation in China, as validated through real-world application? By integrating evidence-based evaluation criteria for clinical value and economic sustainability, which were preliminarily established through 6 criteria derived from our prior DCE study (8), the proposed system seeks to standardize MDs selection processes and enhance transparency in resource utilization under China's evolving healthcare financing policies.

## 2 Materials and methods

### 2.1 MCDA

**Figure 1:**
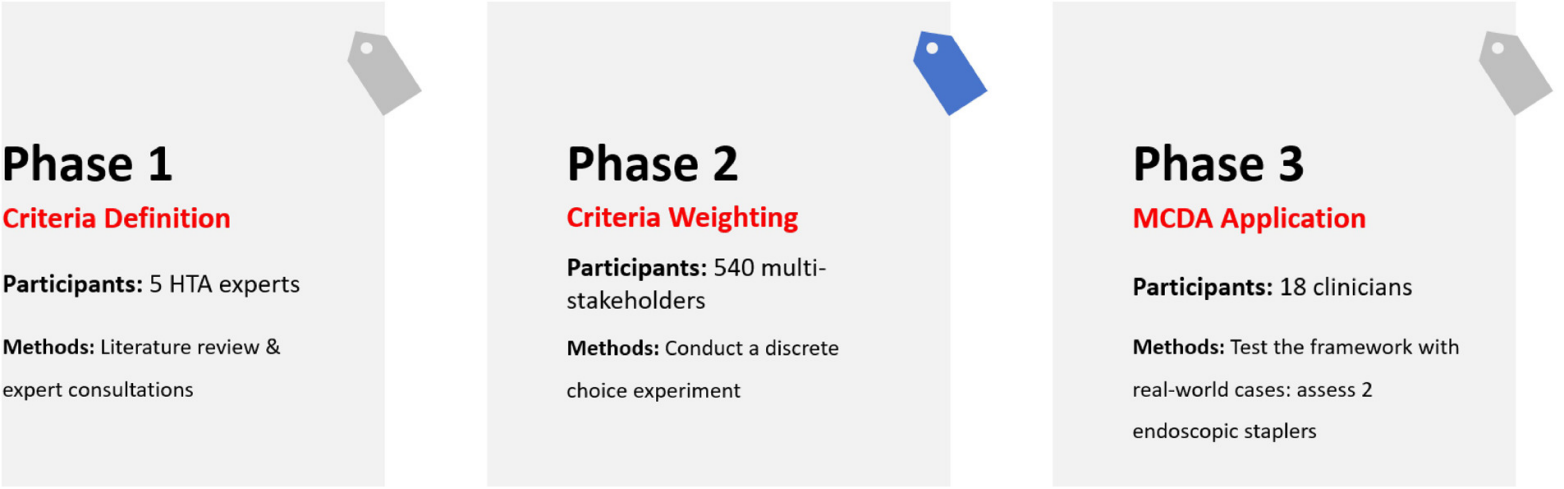
Phases of the MCDA study.

Multicriteria decision analysis structured decision-making by establishing explicit criteria with systematically assigned scores and weights that reflect their relative importance. We constituted a custom weighted sum model in this study. While methodological variations exist across MCDA implementations, four core components consistently emerge: (a) contextual framing of the decision problem; (b) criterion selection and definition; (c) scoring alternatives based on predefined metrics; and (d) weight determination through systematic prioritization (11, 12). To operationalize these principles, our study employed a discrete choice experiment (DCE), a empirical technique validated for preference quantification in healthcare (13, 14). The process of this MCDA study is shown in Figure 1.

**Figure 2:**
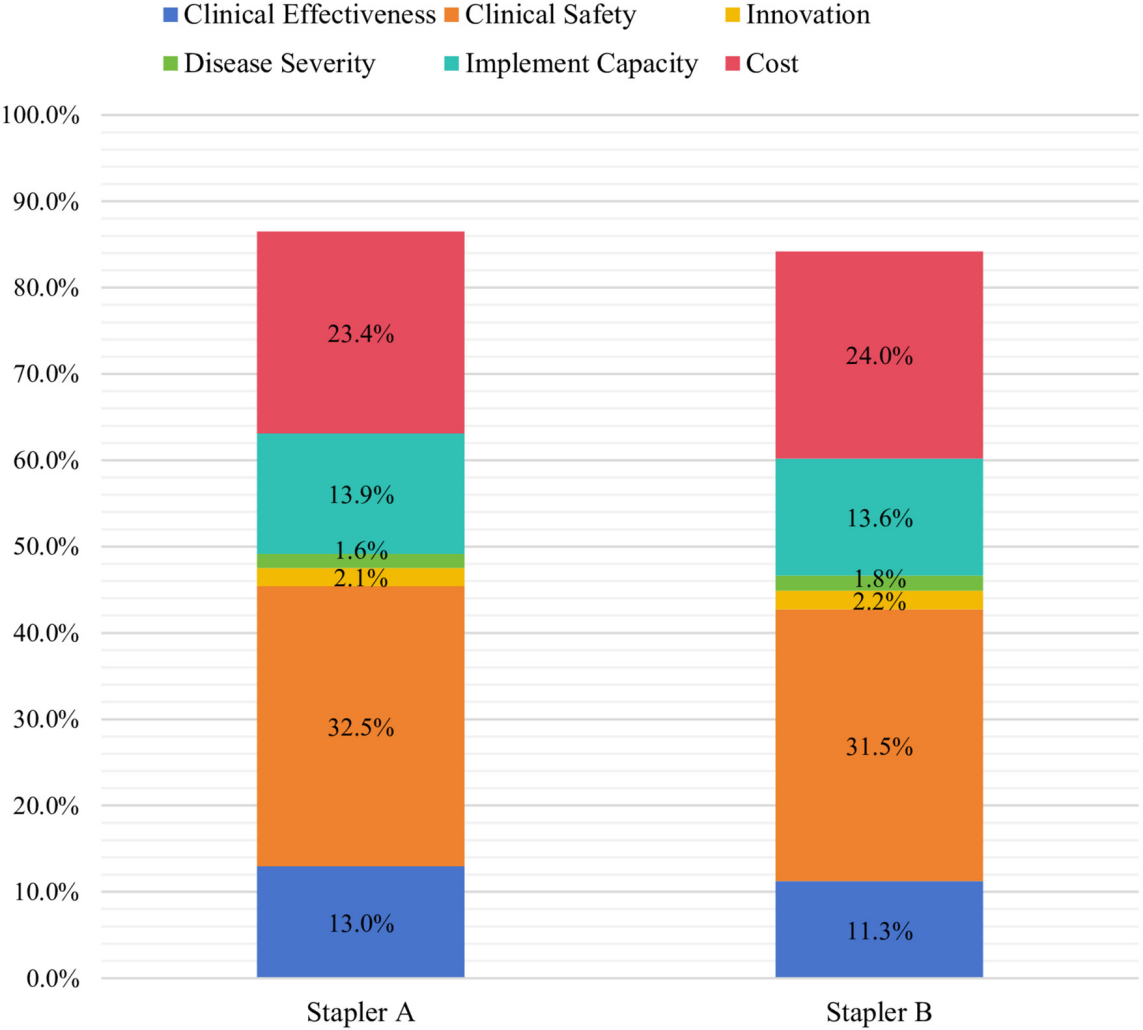
Overall MCDA scores for endoscopic linear cutting staplers. *Stapler A: 95%CI (83.1%, 89.9%); Stapler B: 95%CI (80.9%, 87.5%).

### 2.2 Criteria identification and definition

The criteria for the MCDA in this study were based on the validated attributes and levels derived from our group's previously published DCE research 8. This approach served to validate and confirm the findings reported in that prior study. Building upon established research foundations, a rapid review of medical devices (MDs) prioritization and assessment literature was conducted to identify supplementary evaluation criteria (7, 15, 16). Comprehensive searches were performed in PubMed, Web of Science, and the Cochrane Library through April 2024, utilizing the following search terms: “medical devices”, “decision making”, “discrete choice experiment”, “Multicriteria decision analysis” and “preference”. Identified criteria were systematically collated and critically reviewed to align with foundational DCE principles: completeness, non-redundancy, feasibility and mutual independence. Each criterion underwent rigorous operational definition to ensure conceptual clarity, direct measurability (excluding proxy indicators), and stakeholder interpretability (12).

To finalize the criteria, structured consultations were conducted with five HTA experts, whose selection followed three eligibility criteria: (a) all experts had ≥5 years of experience in health technology assessment; (b) the expert panel included specialists from three key domains (health economics, clinical medicine and health policy) to ensure multi-dimensional perspectives; (c) experts were affiliated with diverse organizations, including a tertiary teaching hospital, and a provincial healthcare security administration, to avoid institutional bias. Consultations were conducted via semi-structured interviews, where experts reviewed the initial criteria list for completeness, non-redundancy, and feasibility. Revisions were integrated iteratively until consensus was reached. After that, 20 participants were randomly sampled to conduct a pilot test to refine six criteria.

### 2.3 Criteria weights

A discrete choice experiment (DCE) was implemented to systematically quantify preferences across multi-stakeholder groups. Participants were purposively sampled based on their roles in pharmaceutical pricing and reimbursement governance, encompassing representatives from national health insurance administrations, tertiary care facilities, HTA committees, clinicians, and patient groups. Adhering to Orme's guidelines for DCE sample adequacy, a minimum cohort of 75 respondents was recruited (17). Given the inclusion of five stakeholder groups, we calculated the required sample size to detect significant preference differences between subgroups. Using G*Power 3.1, assuming a medium effect size (f^2^ = 0.15), *α* = 0.05, and power = 0.80, the minimum sample size per subgroup was estimated at 90. To account for potential invalid responses (e.g., inconsistent answers in the validation set), we oversampled by 10%, resulting in a target sample size of 540 (90 respondents × 5 groups × 1.2). The final valid sample (*n* = 540) exceeded this target, ensuring sufficient power for both overall and subgroup analyses.

Based on the finalized 6 criteria, a D-efficiency design was generated to maximize attribute-level balance while minimizing cognitive burden (18). The design generated 27 different choice tasks, partitioned into three balanced blocks of nine scenarios each. Block assignments were randomized across participants to mitigate order effects. To assess internal consistency, the No.5 choice set within each block was replicated as a verification set (designated set No.10) and integrated into the survey questionnaire. As a result, 48 respondents with inconsistent answers were excluded.

The DCE analysis utilized both Mixed Logit Model and Conditional Logit Model, with final model selection informed by comparative evaluation of Akaike (AIC) and Bayesian (BIC) information criteria (19).

Relative importance (RI) metrics were computed to quantify the maximum marginal contribution of individual criteria to preference formation, normalized to a 100% scale across all evaluated attributes. This metric reflects both stakeholder prioritization patterns and the proportional influence of specific criteria on trade-off decisions (20). For *n* operationalized criteria, RI values were derived from standardized regression coefficients (*β*) using the following equation:

$$RI_{k}=\frac{max\beta_{k}-min\beta_{k}}{\sum_{k=1}^{k}\;(max\beta_{k}-min\beta_{k})}$$

$\beta_{k}$: coefficient.

### 2.4 Application of the MCDA model

Preference data obtained from multi-stakeholder groups in the primary DCE were integrated into a MCDA framework to estimate (a) which among the approved implantable medical devices multi-stakeholders would choose and (b) the impact of each criteria in driving this decision.

The MCDA framework for MDs was applied to access two different endoscopic linear cutting staplers at Jiangsu Provincial People's Hospital in November 2024. Participating clinicians were from gastrointestinal and thoracic surgery departments with prior experience using endoscopic linear cutting staplers.

The consistency of scoring results for the two staplers was accessed using the coefficient of variation (CV). CV < 0.25 was considered indicative of high agreement among the clinicians. Given that the clinicians were from two distinct departments (gastrointestinal surgery and thoracic surgery), we perform a two-sample *t*-test to statistically analyze the average scores assigned to each criterion by the two groups. *P* > 0.05 would indicate that the clinicians scored the criteria based on similar standards.

## 3 Results

### 3.1 Participant characteristics

From May 11 to June 24, 2024, a total of 588 participants completed the survey questionnaire after excluding 48 individuals who failed to meet the validation set (set No. 10 in all survey versions). Among the valid respondents, 12% held decision-making positions within national, provincial, and municipal healthcare security administrations, overseeing medical device tendering and bidding processes, national drug reimbursement list management, and medical insurance payment systems (Table 1). Hospital administrators comprised personnel from medical insurance, medical affairs, and medical device pricing departments, responsible for overseeing medical device management processes within healthcare institutions. Nationally recognized HTA experts accounted for 12% of respondents. Approximately one-quarter (27%) of respondents held positions equivalent to deputy chief physician or higher in specialized medical fields including orthopedics, general surgery, thoracic surgery, neurosurgery, and cardiovascular medicine. The remaining participants represented diverse occupational backgrounds.

**Table 1:** Demographic characteristics in DCE (*n* = 540).

Characteristics	*N*	%
Stakeholder		
Decision-makers	76	14%
HTA experts	65	12%
Hospital administrators	102	19%
Medical doctors	146	27%
Citizens	151	28%
Gender		
Female	135	25%
Male	405	75%
Average age	43	range 22–67 year

### 3.2 Criteria identification and definition

Following a comprehensive review of previously published MDs evaluation criteria, we conducted structured consultations with five HTA experts to finalize 6 criteria: clinical effectiveness, clinical safety, innovation, disease severity, implementation capacity, and cost (Table 2 were placed at the end of the file).

**Table 2:** Multicriteria decision analysis criteria and weights.

Criteria	Definition	Criteria weighting (%)	Criteria categories	Category weight (%)
Clinical Effectiveness	The enhancement of patients’ health outcome following therapy	15.07%	Enhancements in short-term outcomes	7.45%
			Enhancements in both short-term and long-term treatment outcomes	15.07%
Clinical safety	The occurrence rate of adverse reactions associated with MDs, along with associated procedural risks	35.45%	1%	17.52%
			8%	35.45%
Innovation	The introduction of upgrades to existing technologies or the expansion of their application scope to new indications	2.54%	Without: Alternative options available	1.26%
			With: Involves upgrades or expanded applications	2.54%
Disease severity	The critical nature of the targeted disease condition, specifically whether it poses a threat to survival	2.44%	Non-life-threatening condition	2.44%
			Life-threatening condition	1.21%
Implement Capacity	Assessed based on the Implement Capacity across three domains: the healthcare system, medical institutions, and clinicians’ proficiency acquisition	16.56%	Limited assurance in implement capacity across all three domains	0.00%
			Demonstrated implement capacity across any two domains	16.56%
			High assurance in implement capacity across all three domains	11.86%
Cost	The cost of the MDs used in every single treatment	27.94%	2,000 yuan	27.94%
			20,000 yuan	17.77%
			50,000 yuan	0.81%

Clinical effectiveness was categorized into two categories: low-level, defined as enhancements in short-term outcomes (e.g., reduction of surgery duration, the amount of surgical bleeding and the length of hospitalization), and high-level, encompassing both short-term and long-term treatment outcome (e.g., reduction of recurrence rates, extended survival, and improved quality of life).

Clinical safety was evaluated using a two-category classification (1% vs. 8% adverse event incidence), revised from an original three-level system (1%, 8%, 15%) derived from a network meta-analysis of stent-related adverse events (15). Clinicians indicated during pilot testing that an 8% adverse event rate represented a realistic threshold in real world therapy, while 15% was deemed implausible. We recruited 4 clinicians from thoracic surgery with ≥3 years of experience in using implantable devices. Participants completed a pilot version of the DCE questionnaire (including the original 3-level safety criteria: 1%, 8%, 15%) followed by a semi-structured interview. All 4 participants agreed that “15% exceeds the maximum acceptable rate in clinical practice for implantable devices”. Based on this consensus, the 15% level was removed.

The cost of MDs per treatment episode were stratified into three categories (2,000yuan/20,000yuan/50,000yuan), informed by pricing distributions of high-value MDs in Nanjing medical insurance list and experts consultation, consistent with the cost category defined in published studies (16).

Disease Severity and Innovation were defined using two categories adapted from a previously published Queensland study (7). Implementation capacity were assessed across three domains: the healthcare system, medical institutions, and clinicians' proficiency acquisition.

A pilot study involving 14 participants was subsequently implemented to examine data quality, assess respondents’ ability to differentiate criteria categories and make trade-offs between them.

### 3.3 Criteria weights

Methodologically, the mixed logit model outperformed the conditional logit model (AIC: 2,342.17 vs. 2,433.34), attributable to its capacity to account for unobserved preference heterogeneity across stakeholders (e.g., decision-makers prioritized cost containment while clinicians emphasized clinical safety).

The mixed logit model revealed that clinical safety (35.45%, 95% CI: 31.28–39.62%) and cost (27.94%, 95% CI: 24.15–31.73%) emerged as the two most critical criteria for evaluating MDs in China, collectively accounting for over 63% of the total weight (Table 2 were placed at the end of the file). This consistency with the published findings of Wan et al. These findings also align with the priorities of China's ongoing DRG/DIP payment reforms, which emphasize cost containment and risk mitigation in healthcare delivery.

Secondary criteria included implementation capacity (16.56%, 95% CI: 13.82%–19.30%) and clinical effectiveness (15.07%, 95% CI: 12.64%–17.50%), with long-term outcomes weighted higher than short-term gains (7.45%). In contrast, innovation (2.54%, 95% CI: 1.89%–3.19%) and disease severity (2.44%, 95% CI: 1.76%–3.12%) received minimal weight, consistent with industry data indicating that 88% of China's Class III medical devices in 2022 were incremental modifications rather than breakthrough technologies (21).

Subgroup preferences across the six criteria (Supplementary Table S2) revealed both convergence and divergence. There was a clear consensus on clinical safety, with all subgroups showing strong and significant preference for high safety levels (all *P* < 0.001), with hospital administrators (RI = 2.714) and medical doctors (RI = 2.147) placing the highest emphasis, while cost was also consistently prioritized across all subgroups (all *P* < 0.001). Notable divergences included: HTA experts valuing disease severity (RI = 0.379, *P* < 0.000) in contrast to hospital administrators who showed a negative association (RI = −0.578, *P* < 0.000); hospital administrators and medical doctors prioritizing moderate implementation capacity (RI = 1.140, 1.150, all *P* < 0.000) while decision-makers devalued high capacity (RI = −0.923, *P* < 0.000), which may suggest that excessively high implementation thresholds (including technical complexity, training costs, etc.) reduce its practical application value; and medical doctors, citizens, and hospital administrators preferring innovation (RI = 0.546, 0.427, 0.528, all *P* < 0.05) unlike HTA experts (RI = 0.106, *P* = 0.293).

### 3.4 MCDA results

In November 2024, a MCDA was conducted at Jiangsu Provincial People's Hospital for two endoscopic linear cutting staplers: a leading imported brand and a domestic brand. Clinicians independently assessed each device across all predefined criteria using a standardized scoring sheet (Supplementary Figure S1). For each stapler, a composite score was generated via the weighted sum method, a validated MCDA framework (22), by multiplying each criterion score by the weight and summing the weighted criterion scores to produce one total score, as demonstrated in Supplementary Figure S1. Individual total scores were then collated to calculate a mean score for each stapler.

The assessment involved 12 thoracic surgeons and 6 gastrointestinal surgeons, all with prior experience using the evaluated products (Table 3). The overall MCDA scores assigned by clinicians were 86.5% for stapler A and 82.4% for stapler B (see Figure 2). The 4.1% score difference between stapler A and B was significant, as stapler A showed lower adverse event rates and lower long-term cost in real-world use.

**Table 3:** The clinicians’ characteristics in MCDA (*n* = 18).

Characteristics	*N*	%
Gender		
Male	18	100%
Education experience		
Ph.D. or above	13	72%
Master	4	22%
Undergraduate	1	6%
Department		
Thoracic surgery	12	67%
Gastrointestinal surgery	6	33%
Year of service	11.6	Range 2–27 year
Average age	38.7	Range 28–51 year

The consistency of scores across the 18 participating clinicians was evaluated using the coefficient of variation (CV). With CV  < 0.25 for two staplers (0.0914 and 0.0909), indicating a high level of agreement among clinicians. An independent two-sample *t*-test was conducted to compare the average scores for each criterion between the thoracic and gastrointestinal surgeons, assuming unequal variances. The results showed no significant differences (*p* > 0.05) across the six criteria, suggesting that the clinicians scored the staplers based on consistent standards.

## 4 Discussion

This study integrates multicriteria decision analysis (MCDA) and discrete choice experiments (DCEs) to establish a stakeholder-driven evaluation framework for implantable medical devices (MDs) within the context of China's healthcare reform. Under China's current policy landscape, two initiatives dominate medical device procurement and reimbursement: (a) Volume-Based Procurement (VBP), which leverages bulk purchasing to negotiate price reductions for high-value MDs while ensuring quality standards, and (b) medical insurance coverage policies and evidence-driven payment adjustments, which dynamically link reimbursement rates to clinical value and budget impact analyses through evidence-based formulary updates. By establishing an MCDA framework to delineate value parameters of medical devices, this methodological approach systematically addresses multidimensional evaluation criteria through structured value assessment.

By explicitly weighting criteria such as clinical safety (35.45%) and cost (27.94%), our model addresses systemic challenges in China's healthcare system, including fragmented procurement practices and overreliance on short-term cost considerations. The framework's emphasis on safety aligns with regulatory mandates from the National Healthcare Security Administration (NHSA) to prioritize risk mitigation in high-value device reimbursement. Meanwhile, the prominence of cost mirrors DRG/DIP's bundled payment structure, which compels hospitals to balance clinical outcomes with stringent budget constraints—a tension less pronounced in systems like Australia's Queensland framework, where clinical benefit alone accounted for 27.2% of weights 7. While Egypt's tool isolates financial evaluation as a separate post-technical phase, prioritizing technical characteristics (29.4%) and country of origin (19.5%) to emphasize manufacturing quality over immediate cost considerations (9).

Notably, innovation received minimal weight (2.54%). The lower weight of innovation in our study does not indicate that innovation is unimportant. This discrepancy stems from China's current innovation landscape: a significant portion of domestically developed medical MDs approved in recent years have been categorized as incremental modifications (e.g., material upgrades in orthopedic implants), with fewer examples of transformative technological breakthroughs. Such trends reduce perceived value in HTA processes, as minor iterations seldom justify premium pricing under DRG/DIP's fixed reimbursement rates. However, this does not negate innovation's importance; rather, it underscores the need for manufacturers to align R&D with China's policy priorities-specifically, devices that demonstrably lower long-term costs (e.g., reducing revision surgeries) or address unmet clinical needs (e.g., pediatric-specific implants).

The proposed MCDA framework could further optimize decision-making processes across two critical dimensions. First, at the macro-policy level, it supports the design and implementation of value-driven procurement strategies, such as VBP and dynamic adjustments to medical insurance payment standards. By explicitly quantifying trade-offs between cost and clinical outcomes, the framework enables policymakers to prioritize devices that align with population health goals while containing systemic expenditures-a key challenge under China's centralized procurement reforms. Second, the framework enhances transparency in hospital-level decision-making. By incorporating standardized criteria, it provides a replicable protocol for device selection and procurement committees, reducing institutional biases and fostering accountability in MDs adoption.

For health policymakers, this study provides a transparent and evidence-based tool that supports budget planning and equitable resource allocation. The alignment with DRG/DIP payment systems ensures that high-value MDs are reimbursed based on clear, standardized criteria, improving efficiency in medical expenditure.

For healthcare institutions, the MCDA framework offers a structured approach to balance clinical priorities with operational constraints. Hospitals, particularly under DRG/DIP payment models, face dual pressures to optimize clinical outcomes while adhering to strict reimbursement caps. By integrating criteria such as implementation capacity and safety, the framework enables procurement committees to standardize device selection processes, reducing subjective biases in MDs adoption.

For medical device manufacturers, the findings highlight the need for a strategic shift toward developing devices that not only demonstrate clinical superiority but also align with cost-effectiveness and implementation feasibility. The lower priority assigned to incremental innovation indicates that small, non-impactful modifications of existing products are insufficient to gain market access. Instead, companies should focus on substantial advancements that lead to significant clinical and economic improvements.

For emerging technologies, where cost may initially be higher but potential long-term gains are substantial. The framework could be adapted by adjusting cost weights to include long-term economic gains like reducing revision surgeries. For example, a disruptive stent with higher upfront cost but 25% lower 5-year complication rates could be re-evaluated using time-dependent cost metrics.

While our model is tailored to China's institutional context, its core criteria—clinical effectiveness, safety, cost, and implementation capacity—align with global HTA priorities (12). Adapting the framework for cross-country use would require adjusting weights to reflect regional priorities: for instance, high-income countries might assign greater weight to innovation, while low- and middle-income countries could emphasize implementation capacity and cost (9). Aligning with ISPOR's MCDA good practices could facilitate such harmonization.

Despite its strengths, the study has some limitations. The sample size, while substantial, is limited to specific stakeholder groups. Its generalizability may be constrained by the overrepresentation of tertiary hospitals (64% of participants), which face different cost pressures than rural facilities. Stakeholder preferences in our study may also be shaped by cultural and institutional biases inherent to China's healthcare system. Institutionally, VBP and DRG/DIP reforms create strong incentives for cost containment, explaining why decision-makers and hospital administrators prioritized cost more heavily than clinicians. Culturally, the emphasis on collective healthcare sustainability may influence citizens' willingness to trade off marginal innovation for lower costs, as reflected in their moderate preference for innovation (Supplementary Table S2). These biases highlight the need for context-specific calibration when applying the framework across diverse healthcare settings.

Additionally, a multicenter validation study across various hospital settings would strengthen the generalizability of the findings. Incorporating real-world evidence (RWE) into periodic weight updates could enhance the framework's responsiveness to real-world performance, particularly for devices with long-term safety profiles. For example, RWE on post-implantation complication rates could refine the clinical safety weight, while data on long-term healthcare utilization could adjust cost weights to reflect lifetime economic impact. Future studies should explore incorporating real-world clinical data to refine the weighting of criteria further.

The framework's applicability beyond implantable MDs should also be tested in other high-value consumables, such as diagnostic technologies, ensuring broader utility in healthcare decision-making.

## 5 Conclusion

This study presents a novel MCDA-based framework for implantable MD evaluation, tailored to China's healthcare reforms. By incorporating structured criteria weighting, stakeholder preferences, and real-world validation, the model offers a transparent, replicable, and adaptable assessment tool for procurement and reimbursement decisions.

For national-level implementation of the framework, it is advisable to collaborate with the National Healthcare Security Administration to embed the framework into dynamic medical insurance payment adjustment mechanisms, linking reimbursement rates to the weighted scores of core criteria (such as clinical safety and cost) to align with DRG/DIP reforms. A periodic review mechanism should also be established to incorporate real-world evidence from national insurance databases, refining criterion weights and enhancing the framework's responsiveness to evolving clinical needs and policy priorities.

The proposed framework exhibits strong versatility beyond MDs. Its core criteria—clinical effectiveness, safety, cost, and implementation capacity—are adaptable to diverse high-value medical technologies like diagnostic imaging systems and cell/gene therapies. With minor adjustments, such as tailoring “implementation capacity” to fit laboratory needs for diagnostics, the framework can effectively evaluate these technologies, enhancing its utility in healthcare decision-making.

Future refinements integrating multicenter data and real-world evidence will further enhance the framework's robustness and scalability, contributing to evidence-based policymaking and sustainable healthcare financing.

## Data Availability

The original contributions presented in the study are included in the article/Supplementary Material, further inquiries can be directed to the corresponding author.

## References

[1] National healthcare security administration. Statistical bulletin of the national healthcare security administration. (2023). Available online at: https://www.nhsa.gov.cn/art/2024/7/25/art_7_13340.html (Accessed July 25, 2024).

[2] National Healthcare Security Administration. The national medical insurance bureau officially launched the three-year action plan for DRG/DIP payment reform. (2021). Available online at: http://www.nhsa.gov.cn/art/2021/11/26/art_104_7413.html (Accessed August 10, 2023).

[3] World Health Organization. Health-topic medical-devices. (2023). Available online at: https://www.emro.who.int/health-topics/medical-devices/index.html (Accessed August 10, 2023).

[4] National Healthcare Security Administration. Pilot plan for centralized procurement and use of drugs. (2019). Available online at: https://www.gov.cn/gongbao/content/2019/content_5361793.htm (Accessed January 1, 2019).

[5] National Healthcare Security Administration. Regulations on medical equipment procurement management. (2019). Available online at: https://www.gov.cn/gongbao/content/2019/content_5442286.htm (Accessed June 6, 2019).

[6] Chuanchao L Xin G. International experience and implications of real-world evidence supporting drug medical insurance access. China Health Insur. (2024) 2024(7):24–32. 10.19546/j.issn.1674-3830.2024.7.003

[7] Howard S Scott IA Ju H McQueen L Scuffham PA. Multicriteria decision analysis (MCDA) for health technology assessment: the Queensland health experience. Aust Health Rev. (2019) 43(5):591–9. 10.1071/AH18042 30205873

[8] Wan B Shen J Chen J Weng L Zhao P Deng Y Quantifying stakeholders’ preference for implantable medical devices in China: a discrete choice experiment. Int J Technol Assess Health Care. (2024) 40(1):e8. 10.1017/S0266462323002799 38221900 PMC10859836

[9] Elezbawy B Fasseeh AN Németh B Gamal M Eldebeiky M Refaat R A multicriteria decision analysis (MCDA) tool to purchase implantable medical devices in Egypt. BMC Med Inform Decis Mak. (2022) 22(1):289. 10.1186/s12911-022-02025-y 36352382 PMC9644459

[10] Khan I Pintelon L Martin H. The application of multicriteria decision analysis methods in health care: a literature review. Med Decis Mak. (2022) 42(2):262–74. 10.1177/0272989X211019040 34166149

[11] Devlin N Sussex J. Incorporating Multiple Criteria in HTA: Methods and Processes. London: Office of Health Economics (2011).

[12] Marsh K IJzerman M Thokala P Baltussen R Boysen M Kaló Z Multiple criteria decision analysis for health care decision making–emerging good practices: report 2 of the ISPOR MCDA emerging good practices task force. Value Health. (2016) 19(2):125–37. 10.1016/j.jval.2015.12.016 27021745

[13] Clark MD Determann D Petrou S Moro D de Bekker-Grob EW. Discrete choice experiments in health economics: a review of the literature. PharmacoEconomics. (2014) 32(9):883–902. 10.1007/s40273-014-0170-x 25005924

[14] Soekhai V de Bekker-Grob EW Ellis AR Vass CM. Discrete choice experiments in health economics: past, present and future. PharmacoEconomics. (2019) 37(2):201–26. 10.1007/s40273-018-0734-2 30392040 PMC6386055

[15] Madhavan MV Kirtane AJ Redfors B Généreux P Ben-Yehuda O Palmerini T Stent-related adverse events >1 year after percutaneous coronary intervention. J Am Coll Cardiol. (2020) 75(6):590–604. 10.1016/j.jacc.2019.11.058 32057373

[16] Lee HJ Bae EY. Eliciting preferences for medical devices in South Korea: a discrete choice experiment. Health Policy. (2017) 121(3):243–9. 10.1016/j.healthpol.2017.01.002 28117075

[17] Orme BK. Getting Started with Conjoint Analysis: Strategies for Product Design and Pricing Research. 4. Manhattan Beach, CA: Research Publishers LLC (2020).

[18] Reed Johnson F Lancsar E Marshall D Kilambi V Mühlbacher A Regier DA Constructing experimental designs for discrete-choice experiments: report of the ISPOR conjoint analysis experimental design good research practices task force. Value Health. (2013) 16(1):3–13. 10.1016/j.jval.2012.08.2223 23337210

[19] Dziak JJ Coffman DL Lanza ST Li R Jermiin LS. Sensitivity and specificity of information criteria. Brief Bioinform. (2020) 21(2):553–65. 10.1093/bib/bbz016 30895308 PMC7299313

[20] Hauber AB González JM Groothuis-Oudshoorn CG Prior T Marshall DA Cunningham C Statistical methods for the analysis of discrete choice experiments: a report of the ISPOR conjoint analysis good research practices task force. Value in Health. (2016) 19(4):300–15. 10.1016/j.jval.2016.04.004 27325321

[21] Dong J Yujun C Xiaoping C. Annual Report on the Data of Medical Device Industry in China (2023). Peking: Social Sciences Academic Press (China) (2023).

[22] Thokala P Duenas A. Multiple criteria decision analysis for health technology assessment. Value in Health. (2012) 15(8):1172–81. 10.1016/j.jval.2012.06.015 23244821

