# Correction: A multi-stakeholder multicriteria decision analysis for implantable medical devices assessment in China

**DOI:** 10.3389/frhs.2025.1695328

**Published:** 2025-09-19

**Authors:** Yizhou Xu, Junjie Wan, Bin Wan, Haixia Ding

**Affiliations:** ^1^ School of Pharmacy, Nanjing Medical University, Nanjing, Jiangsu, China; ^2^ School of International Pharmaceutical Business, China Pharmaceutical University, Nanjing, Jiangsu, China; ^3^ Department of Health Insurance Management, The First Affiliated Hospital with Nanjing Medical University, Nanjing, Jiangsu, China

**Keywords:** medical devices, multicriteria decision analysis, discrete choice experiment, technology assessment, preference, China

**Author affiliation**

**Erroneously assigned**

Adding:

Affiliation [Department of Health Insurance Management, The First Affiliated Hospital with Nanjing Medical University, No.300 Guangzhou Road, Gulou District, Nanjing 210029, Jiangsu, P.R. China] was omitted for author [Haixia Ding]. This affiliation has now been added for author [Haixia Ding].

The original version of this article has been updated.

