# Supplementary material for: A multi-stakeholder multicriteria decision analysis for implantable medical devices assessment in China
Source: Front Health Serv. 2025 Aug 29;5:1650709. doi: 10.3389/frhs.2025.1650709 (PMC12426139; doi:10.3389/frhs.2025.1650709)
Supplement: Supplementary file 1 [file Table1.docx]

SUPPLEMENTARY MATERIAL

A multi‑stakeholder multicriteria decision analysis for implantable medical devices assessment in China

1. **Table S1.** Multi-stakeholders’ preference for high-value MDs: main effects of mixed logit model results
2. **Table S2.** Relative importance based on the results of main effects of mixed logit model by subgroups
3. **Table S3.** Stapler scoring sheet

**Table S1. Multi-stakeholders’ preference for high-value MDs: main effects of mixed logit model results**

| Attributes | Levels | Coefficient | SE | Z | P value | 95% CI | |
| --- | --- | --- | --- | --- | --- | --- | --- |
| Clinical Effectiveness | Low (reference) |  |  |  |  |  |  |
|  | High | 0.974 | 0.119 | 8.16 | <0.001 | 0.740 | 1.207 |
| Clinical Safety | Low (reference) |  |  |  |  |  |  |
|  | High | 2.290 | 0.192 | 11.95 | <0.001 | 1.914 | 2.665 |
| Innovation | Without (reference) |  |  |  |  |  |  |
|  | With | 0.164 | 0.082 | 1.99 | 0.047 | 0.002 | 0.326 |
| Disease Severity | Low (reference) |  |  |  |  |  |  |
|  | High | -0.157 | 0.098 | -1.60 | 0.109 | -0.350 | 0.035 |
| Implement Capacity | Low (reference) |  |  |  |  |  |  |
|  | Middle | 1.070 | 0.188 | 5.68 | <0.001 | 0.701 | 1.439 |
|  | High | 0.769 | 0.142 | 5.42 | <0.001 | 0.491 | 1.048 |
| Cost |  | -0.0000361 | <0.001 | -10.19 | <0.001 | <0.001 | <0.001 |

*None

**Table S2. Relative importance based on the results of main effects of mixed logit model by subgroups**

| **Criteria categories** | **RI** | **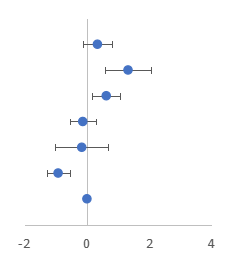95%CI** | |  |  | **P-value** |
| --- | --- | --- | --- | --- | --- | --- |
| **Decision-makers** |  |  |  |  |  |  |
| Clinical Effectiveness-High | 0.336 | -0.124 | 0.797 |  |  | 0.153 |
| Clinical Safety-High | 1.315 | 0.594 | 2.037 |  |  | 0.000 |
| Innovation-With | 0.619 | 0.165 | 1.073 |  |  | 0.008 |
| Disease Severity-High | -0.134 | -0.543 | 0.276 |  |  | 0.522 |
| Implement Capacity-Moderate | -0.166 | -1.010 | 0.677 |  |  | 0.699 |
| Implement Capacity-High | -0.923 | -1.292 | -0.555 |  |  | 0.000 |
| Cost | 0.000 | 0.000 | 0.000 |  |  | 0.000 |
|  |  |  |  |  |  |  |
|  |  |  | 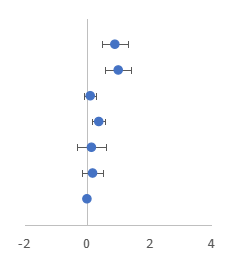 |  |  |  |
| **HTA experts** |  |  |  |  |  |  |
| Clinical Effectiveness-High | 0.894 | 0.485 | 1.302 |  |  | 0.000 |
| Clinical Safety-High | 1.002 | 0.589 | 1.415 |  |  | 0.000 |
| Innovation-With | 0.106 | -0.092 | 0.304 |  |  | 0.293 |
| Disease Severity-High | 0.379 | 0.175 | 0.582 |  |  | 0.000 |
| Implement Capacity-Moderate | 0.144 | -0.332 | 0.620 |  |  | 0.554 |
| Implement Capacity-High | 0.181 | -0.161 | 0.523 |  |  | 0.300 |
| Cost | 0.000 | 0.000 | 0.000 |  |  | 0.000 |
|  |  |  |  |  |  |  |
|  |  |  |  |  |  |  |
| **Hospital administrators** |  |  | 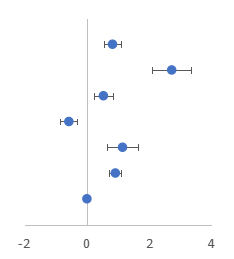 |  |  |  |
| Clinical Effectiveness-High | 0.819 | 0.535 | 1.102 |  |  | 0.000 |
| Clinical Safety-High | 2.714 | 2.081 | 3.346 |  |  | 0.000 |
| Innovation-With | 0.528 | 0.222 | 0.834 |  |  | 0.001 |
| Disease Severity-High | -0.578 | -0.845 | -0.311 |  |  | 0.000 |
| Implement Capacity-Moderate | 1.140 | 0.647 | 1.633 |  |  | 0.000 |
| Implement Capacity-High | 0.909 | 0.712 | 1.107 |  |  | 0.000 |
| Cost | 0.000 | 0.000 | 0.000 |  |  | 0.000 |
|  |  |  |  |  |  |  |
|  |  |  | 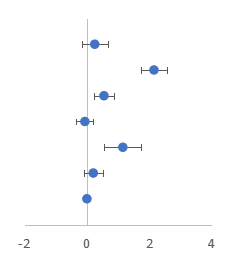 |  |  |  |
| **Medical doctors** |  |  |  |  |  |  |
| Clinical Effectiveness-High | 0.251 | -0.165 | 0.666 |  |  | 0.237 |
| Clinical Safety-High | 2.147 | 1.732 | 2.561 |  |  | 0.000 |
| Innovation-With | 0.546 | 0.238 | 0.854 |  |  | 0.001 |
| Disease Severity-High | -0.066 | -0.341 | 0.210 |  |  | 0.641 |
| Implement Capacity-Moderate | 1.150 | 0.559 | 1.742 |  |  | 0.000 |
| Implement Capacity-High | 0.204 | -0.105 | 0.513 |  |  | 0.195 |
| Cost | 0.000 | 0.000 | 0.000 |  |  | 0.000 |
|  |  |  |  |  |  |  |
|  |  |  |  |  |  |  |
| **Citizens** |  |  | 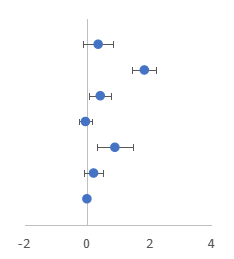 |  |  |  |
| Clinical Effectiveness-High | 0.359 | -0.117 | 0.836 |  |  | 0.139 |
| Clinical Safety-High | 1.838 | 1.448 | 2.228 |  |  | 0.000 |
| Innovation-With | 0.427 | 0.079 | 0.776 |  |  | 0.016 |
| Disease Severity-High | -0.048 | -0.263 | 0.168 |  |  | 0.664 |
| Implement Capacity-Moderate | 0.894 | 0.319 | 1.470 |  |  | 0.002 |
| Implement Capacity-High | 0.213 | -0.079 | 0.505 |  |  | 0.152 |
| Cost | 0.000 | 0.000 | 0.000 |  |  | 0.002 |
|  |  |  |  |  |  |  |
|  |  |  |  |  |  |  |

*RI=relative importance; HTA=health techonology assessment.

**Table S3.** Stapler scoring sheet

**Stapler A**

**Please check "**✔**" in the appropriate box under each indicator category.**

| **Criteria** | **Definition** | **Criteria category** | | | **Weighted scores** |
| --- | --- | --- | --- | --- | --- |
| Clinical Effectiveness | The improvement of patients’ health outcome after treatment and short-term and long-term therapeutically effectiveness. | Low |  | High | 15.07% |
|  |  |  |  | ✔ |  |
| Clinical Safety | Adverse event incidence rate of the MDs and operational risk. | Low |  | High | 35.45% |
|  |  |  |  | ✔ |  |
| Innovation | New iteration or new indication of existing technology. | Without |  | With | 1.26% |
|  |  | ✔ |  |  |  |
| Disease Severity | Whether the targeted disease was life-threatening or not. | Low |  | High | 1.21% |
|  |  |  |  | ✔ |  |
| Implement Capacity | Implementation capacity was assessed from three different sector, the health system, the medical institute and medical doctors’ learning curve. | Low | Moderate | High | 16.56% |
|  |  |  | ✔ |  |  |
| Cost | Cost per treatment using the stapler | 2000  yuan | 20000  yuan | 50000  yuan | 17.77% |
|  |  |  | ✔ |  |  |

Weighted scores：87.32%
